# Supplementary material for: MicroRNA Profiles of Maternal and Neonatal Endothelial Progenitor Cells in Preeclampsia
Source: Int J Mol Sci. 2021 May 18;22(10):5320. doi: 10.3390/ijms22105320 (PMC8158476; doi:10.3390/ijms22105320)
Supplement: Supplementary file 1 [file ijms-22-05320-s001.zip › Supplemental Figures_Tables_proof_final.pdf]

Appendix A

Supplemental Methods S1

MicroRNA-Seq experiment
Library generation: 500 ng of total RNA (including small RNA) were utilized per sample as input for cDNA library generation using 'NEBNext® Multiplex Small RNA Library Prep Kit for Illumina® 96 rxns; Index Primers 1-48' (E7560S; New England Biolabs). All steps were performed as recommended in user manualE7560 (Version 1.0_08-2016; NEB) except that all reactions were downscaled to 1/2 of initial volumes. cDNA libraries were barcoded by single indexing approach, using contained 'NEBNext Multiplex Oligos for Illumina (Index Primer 1 – 48). All generated cDNA libraries were amplified by 12 cycles of final pcr. Fragment length distribution of individual libraries was monitored using 'Bioanalyzer DNA 1000 Assay' (5067-1504; Agilent Technologies). Quantification of libraries was additionally performed by use of the 'Qubit® dsDNA HS Assay Kit' (Q32854; ThermoFisher Scientific). Equal molar amounts of twenty-four differently barcoded smallRNA libraries in total were pooled for one sequencing run and were subjected to size selection. Mature miRNA libraries appeared as a peak at 147 bp (corresponding to a 21 nucleotide insert flanked by illumina adapters). Size selection using the BluePippin was performed as described in the "Blue Pippin DNA Size Selection System Operations Manual Software v6.23 Cassette Definition Set 22" using the following settings and parameters: Cassette: '3% agarose'; Standards: 'Internal'; Programming Mode: 'Tight'; Target base pair value: '146'. 30 µl of sample were combined with 10 µl of DNA marker F and were loaded on one well of the 3% agarose cassette (Biozyme # BDQ3003). After elution, 45 µl of sample were reduced to 20µl (speedvac) and 1µl was subjected to a 'Bioanalyzer DNA HS Assay' (5067-4626; Agilent Technologies).
Sequencing run: An aliquot of the pooled size-selected library was denatured with NaOH and finally diluted to 1.5pM according to the Denature and Dilute Libraries Guide (Document # 15048776 v02; Illumina). 1.3 ml of denatured pool were loaded on an Illumina NextSeq 550 sequencer using a High Output Flowcell for 75bp single reads (#FC-404-2005; Illumina).
Raw data processing and quality control:
BCL files were converted to FASTQ files using bcl2fastq Conversion Software version 2.17.1.14 (Illumina). The FASTQ files were adapter and quality trimmed using Trim Galore! (version 0.4.1) with default settings as described in the User Guide except for the setting of the quality cutoff (-q/--quality) which was set to a Phred score of 15 and the length cutoff (--length) which was set to 6bp. Trim Galore! used Cutadapt (version 1.9.1) as subroutine.
Quality control of FASTQ files was performed by FastQC (version 0.11.4) before and after trimming.
Data import to StrandNGS: Trimmed FASTQ files were imported to StrandNGS software (version 3.1). A Small RNA Alignment experiment type was created according to the following settings: Build: Human hg19 (UCSC); Sequencing Platform: Illumina; Library layout: Directional Single End.
The alignment was conducted with following parameters: Number of mismatches allowed: 1; Minimum match length: 10; Maximum number of matches to be reported per read: 5; Ignore reads with more than 5 valid matches; Fixed Trimming was inactivated; Trim 3' end with average quality less than 10.
For quantification and analysis, a Small RNA Analysis Experiment type was created, according to the following settings: Build: Human hg19; Transcript Annotation: RefSeq (2015.10.05); Small RNA Annotation: SmallRNA SmallRNA (2012.01.15); Sequencing Platform: Illumina; Library layout: Directional Single End. Quantification was performed using Quantile normalization algorithm (including partial reads). Read counts were thresholded to 1. No baseline transformation was conducted.

Supplemental Table 1. Global overview of regulated miRNAs which passed Benjamini-Hochberg-correction. Fold change refers to the reference group (cord blood = 1).

miRNA	Fold Change	p-value
**Cord blood vs. Maternal blood ECFC from control in P5**		
hsa-miR-4485	7.77	0.03
hsa-miR-4284	3.67	0.03
hsa-miR-328	2.35	0.03
hsa-miR-365a-5p	2.17	0.04
**Cord blood vs. Maternal blood ECFC from preeclampsia in P3**		
hsa-miR-3938	-3.68	0.002
hsa-miR-551a	-3.51	0.02
hsa-miR-196b-5p	-2.34	0.03
hsa-miR-338-5p	4.01	0.003
hsa-miR-1250	3.98	0.03
hsa-miR-338-3p	3.45	0.003
**Cord blood vs. Maternal blood ECFC from preeclampsia in P5**		
hsa-miR-551a	-9.89	0.02
hsa-miR-3938	-9.55	0.001
hsa-miR-556-5p	-6.36	0.02
hsa-miR-2467-5p	-5.19	0.001
hsa-miR-196b-5p	-5.03	0.02
hsa-miR-196b-5p	-5.03	0.02
hsa-miR-26a-1-3p	-2.92	0.01
hsa-miR-4687-3p	-2.52	0.02
hsa-miR-338-5p	13.32	0.01
hsa-miR-4485	5.76	0.04
hsa-miR-664-3p	3.79	0.02
hsa-miR-451a	3.71	0.04
hsa-let-7b-3p	3.30	0.02
hsa-miR-4497	2.89	0.04
hsa-miR-23c	2.67	0.04
hsa-miR-3615	2.65	0.01
hsa-miR-193b-3p	2.53	0.02
hsa-miR-193b-5p	2.52	0.02
hsa-miR-2682-5p	2.46	0.02
hsa-miR-574-3p	2.34	0.02
hsa-miR-23a-5p	2.29	0.01
hsa-let-7b-5p	2.28	0.04
hsa-miR-365b-3p	2.26	0.02
hsa-miR-365a-3p	2.26	0.04
hsa-miR-140-3p	2.22	0.02
hsa-miR-328	2.17	0.02
hsa-miR-4479	2.11	0.04

Supplemental Table 3A. Primer sequences for target genes.

Primer		Sequence 5´ … 3´
TFRC	forward	AGA CTT TGG ATC GGT TGG TG
	reverse	TTA AAT GCA GGG ACG AAA GG
ANGPTL7	forward	CTG CAC AGA CTC CAA CCT CA
	reverse	GCC ATC CAG GTG CTT ATT GT
RNA18S1	forward	ACA TCC AAG GAA GGC AGC AG
	reverse	TTT TCG TCA CTA CCT CCC CG
NOSTRIN	forward	CCT GGG GAA AGT GTG TGA GT
	reverse	CTT CTC CAG CTC CAG AAT GC
HMGA2	forward	n.A.*
	reverse	n.A.*

*= The sequence was not provided by the manufacturer (Thermo Fisher Scientific).

Supplemental Table 3B. Primer sequences for the differently expressed miRNA.

Assay name	microRNA sequence (Qiagen)
RNU6	RNU6-6P RNA, U6 small nuclear 6, pseudogene
1270	5'CUGGAGAUAUGGAAGAGCUGUGU´3

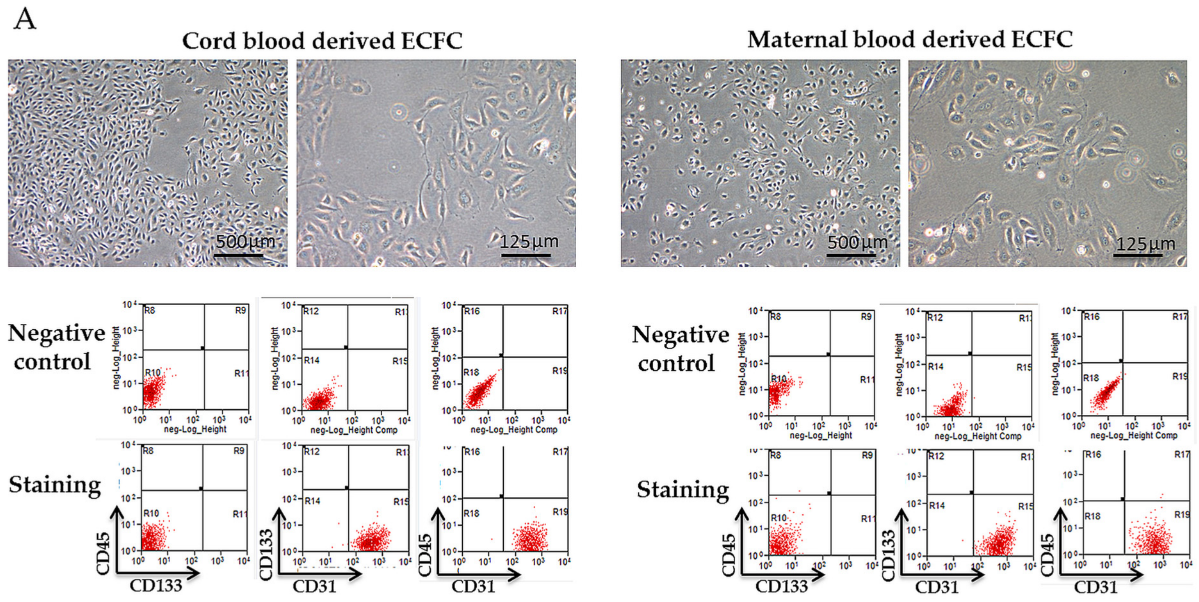

B

	Cord blood derived ECFC			Maternal blood derived ECFC		
	Healthy pregnancy (n = 6)	Preeclamptic pregnancy (n = 6)	p-value	Healthy pregnancy (n = 6)	Preeclamptic pregnancy (n = 6)	p-value
Time to first ECFC colony (days)	7.6 ± 1.6	10.8 ± 2.6	0.04	13.2 ± 4.3	15.2 ± 1.7	0.31
Total number of ECFC colonies (n)	8.2 ± 2.3	10.2 ±6.8	0.50	1.5 ± 0.8	1.75 ± 1.3	0.69

Supplemental Figure S1. (A) Flow cytometric analyses and ECFC phenotype. (B) ECFC colony formation and appearance of Cord blood and Maternal blood derived ECFC.

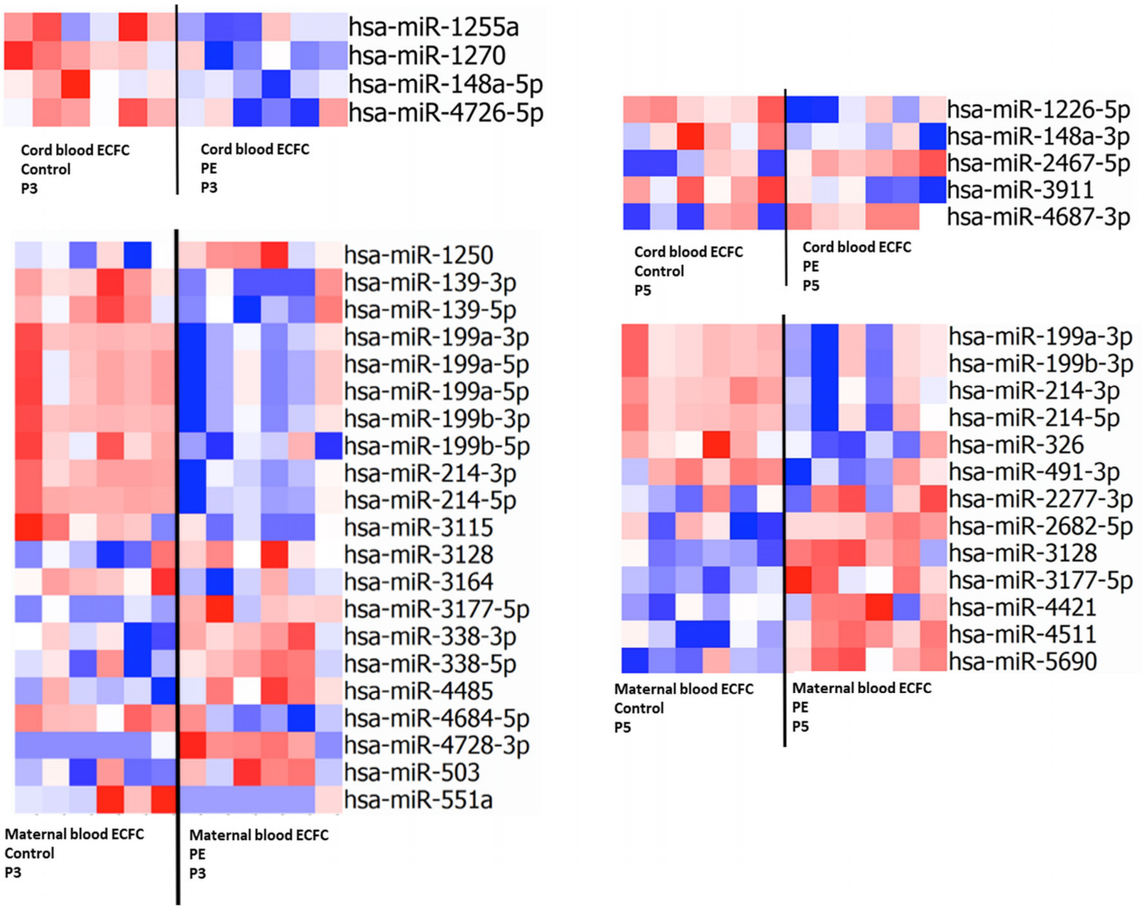

Supplemental Figure S2A: Heat maps of significantly regulated miRNAs in ECFC of controls compared to preeclampsia. Visualisation of inspect of expression changes of candidate miRNAs. PE: preeclamptic pregnancy, P3: passage 3, P5: passage 5.

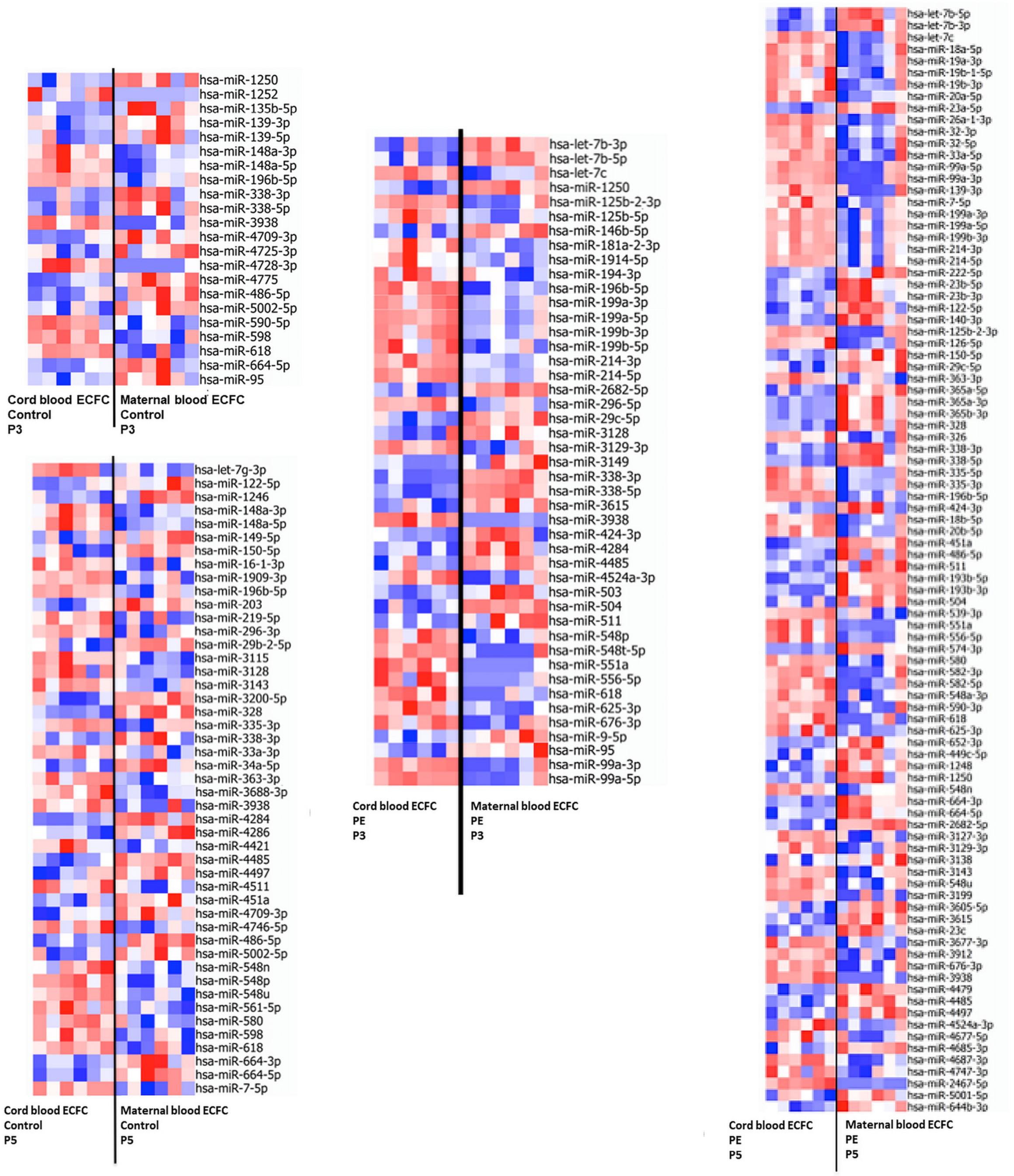

Supplemental Figure S2B: Heat maps of significantly regulated miRNAs in ECFCs of Cord blood vs. Maternal blood. Visualization of inspect of expression changes of candidate miRNAs. PE: preeclamptic pregnancy, P3: passage 3, P5: passage 5.

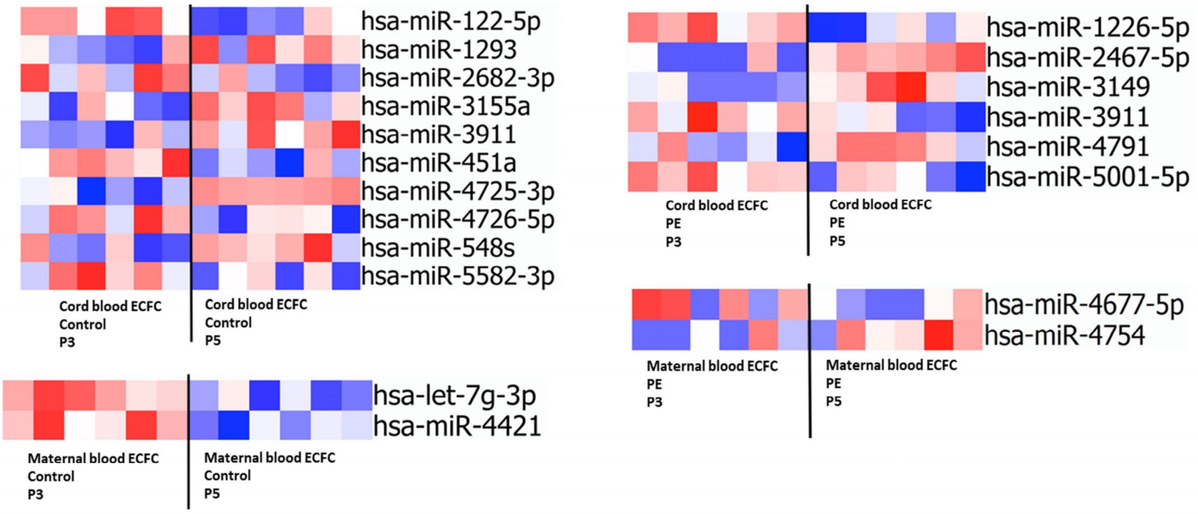

Supplemental Figure S2C: Heat maps of significantly regulated miRNAs in ECFC of P3 vs. P5. Visualization of inspect of expression changes of candidate miRNAs. PE: preeclamptic pregnancy, P3: passage 3, P5: passage 5.

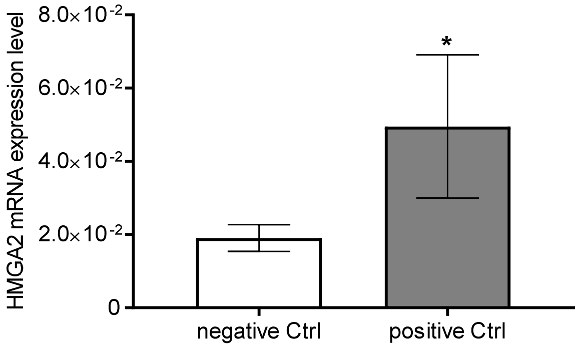

Supplemental Figure S3: qRT-PCR analysis of *HMGA2* expression level after transfection with *mir*Vana™ miRNA inhibitor let-7c positive control (positive Ctrl) and *mir*Vana™ miRNA inhibitor negative control #1 (negative Ctrl). $p<0.05$.

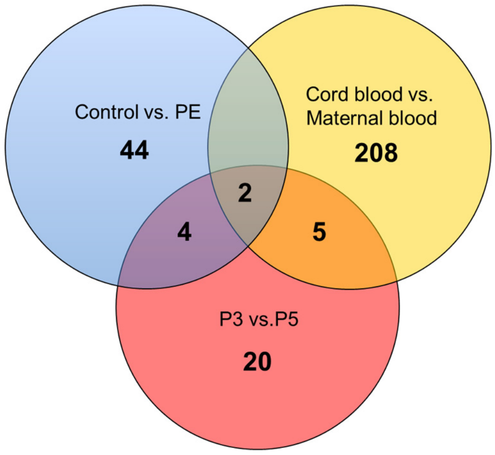

Supplemental Figure S4: Venn diagram showing the overlap between miRNA expression changes in the different set of samples, namely Control vs. PE (preeclampsia), Cord blood vs. Maternal blood and P3 vs. P5. Two miRNAs showed different expression changes in all entities (hsa-miR-2467-5p; hsa-miR-4421).

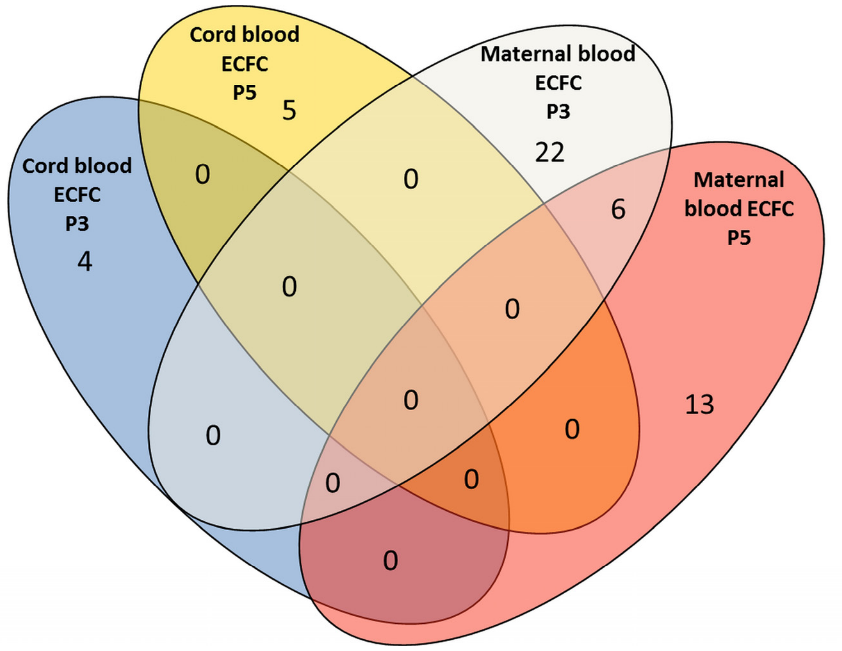

PE: preeclampsia; P3: passage 3; P5: passage 5.

Supplemental Figure S5: Venn diagram showing the overlap between miRNA expression changes in the different set of samples of significantly regulated miRNAs in ECFC of controls compared to PE (preeclampsia). P3: passage 3; P5: passage 5.

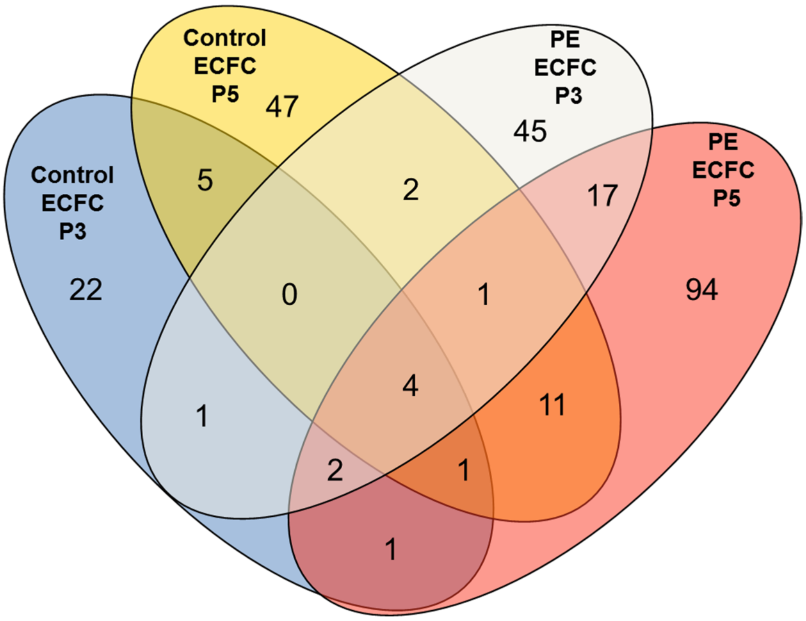

Supplemental Figure S6: Venn diagram showing the overlap between miRNA expression changes in the different set of samples of significantly regulated miRNAs in ECFC of Cord blood vs. Maternal blood. PE: preeclampsia; P3: passage 3; P5: passage 5.

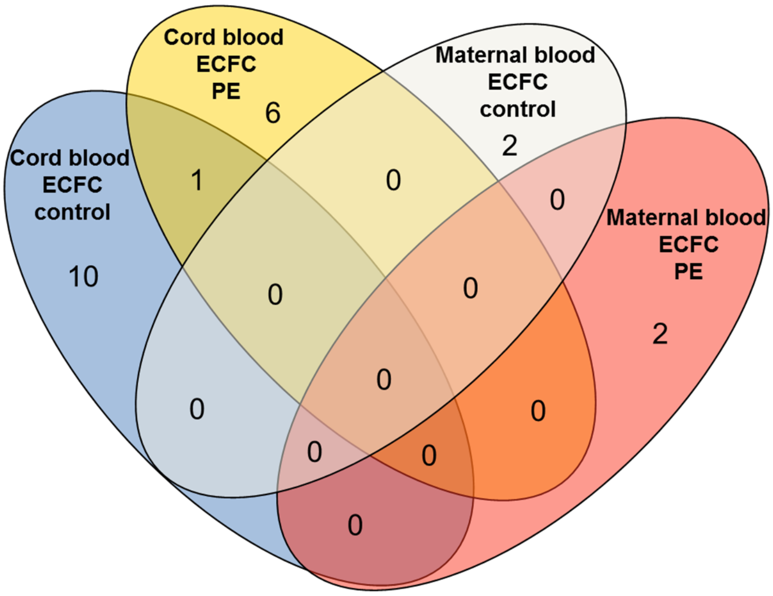

Supplemental Figure S7: Venn diagram showing the overlap between miRNA expression changes in the different set of samples of significantly regulated miRNAs in ECFC of P3 vs. P5. P3: passage 3; P5: passage 5.
